# Visual motion-sensitive neurons in the bumblebee brain convey information about landmarks during a navigational task

**DOI:** 10.3389/fnbeh.2014.00335

**Published:** 2014-09-24

**Authors:** Marcel Mertes, Laura Dittmar, Martin Egelhaaf, Norbert Boeddeker

**Affiliations:** Department of Neurobiology, Center of Excellence ‘Cognitive Interaction Technology’ (CITEC), Bielefeld UniversityBielefeld, Germany

**Keywords:** optic-flow, insect, motion sensing, homing, electrophysiology

## Abstract

Bees use visual memories to find the spatial location of previously learnt food sites. Characteristic learning flights help acquiring these memories at newly discovered foraging locations where landmarks—salient objects in the vicinity of the goal location—can play an important role in guiding the animal's homing behavior. Although behavioral experiments have shown that bees can use a variety of visual cues to distinguish objects as landmarks, the question of how landmark features are encoded by the visual system is still open. Recently, it could be shown that motion cues are sufficient to allow bees localizing their goal using landmarks that can hardly be discriminated from the background texture. Here, we tested the hypothesis that motion sensitive neurons in the bee's visual pathway provide information about such landmarks during a learning flight and might, thus, play a role for goal localization. We tracked learning flights of free-flying bumblebees (*Bombus terrestris*) in an arena with distinct visual landmarks, reconstructed the visual input during these flights, and replayed ego-perspective movies to tethered bumblebees while recording the activity of direction-selective wide-field neurons in their optic lobe. By comparing neuronal responses during a typical learning flight and targeted modifications of landmark properties in this movie we demonstrate that these objects are indeed represented in the bee's visual motion pathway. We find that object-induced responses vary little with object texture, which is in agreement with behavioral evidence. These neurons thus convey information about landmark properties that are useful for view-based homing.

## Introduction

Bees, ants, and wasps are exquisitely able to find back to important places like their nest or valuable food sources using several navigational strategies including path integration, route following and landmark navigation (Menzel and Müller, 1996; Collett and Collett, 2002; Collett et al., 2006; Zeil et al., 2009; Zeil, 2012). Landmarks are salient objects that provide reliable information about the goal location (Gillner et al., 2008). Information about the landmark constellation around the goal is presumably acquired and stored during learning flights where the bees face the goal and perform highly stereotyped arcs and loops at and around the goal location (Lehrer, 1993; Collett and Zeil, 1996; Zeil et al., 1996; Hempel Ibarra et al., 2009; Collett et al., 2013; Philippides et al., 2013). Since honeybees can employ distance information and are even able to find a goal between camouflaged landmarks that carry the same texture as the background (Dittmar et al., 2010), it has been proposed that the insects memorize the motion pattern on their eyes (“optic flow”) generated during the learning flights (Zeil, 1993b; Dittmar et al., 2010). However, distance information can only be gained and camouflaged landmarks can only be detected in the optic flow patterns, if the movements of the bee contain a sufficiently pronounced translational component: Exclusively during translational self-motion, close objects move faster on the retina than objects further away, allowing the animal to potentially use the resulting motion parallax cues to infer the distance to objects. In contrast, during pure rotations of the animal the perceived optic flow is independent of object distance, and all objects move with the same speed on the retina (Koenderink, 1986). However, specific combinations of rotatory and translatory self-motion—for instance, when the animal circles around a pivot point while fixating it—may generate an optic flow pattern, where the retinal images of objects before and behind the pivot point move in opposite directions (Collett and Zeil, 1996; Zeil et al., 1996).

Flying hymenopterans, such as honeybees, bumblebees, and wasps (Boeddeker et al., 2010, submitted manuscript; Braun et al., 2012; Zeil, 2012) as well as various fly species (Schilstra and van Hateren, 1999; van Hateren and Schilstra, 1999; Mronz and Lehmann, 2008; Braun et al., 2010; Geurten et al., 2010; Kern et al., 2012; van Breugel and Dickinson, 2012) show a saccadic flight and gaze strategy, where translational and rotational changes in gaze appear to be largely separated. This distinguishing flight characteristic has been suggested to facilitate the processing of depth information from motion parallax cues (Egelhaaf et al., 2012; Schwegmann et al., 2014) by minimizing the time of flight during which rotations occur by performing extremely fast body and head rotations (“saccades”). Within the longer segments of flight, the intersaccades, the gaze is kept straight. Therefore, the animals perceive almost purely translational optic flow. The hypothesis that the nervous system may indeed use optic flow during intersaccadic translatory flight to gather spatial information is supported by the finding that motion sensitive neurons in the third visual neuropile of flies have been found to vary with the three-dimensional structure of the environment during translatory intersaccadic flight (Boeddeker et al., 2005; Kern et al., 2005, 2006; Karmeier et al., 2006; Hennig and Egelhaaf, 2012; Liang et al., 2012).

All the above-mentioned electrophysiological studies in flies, which investigated how environmental information is represented by motion sensitive neurons during intersaccadic intervals, were done for spontaneous flights, i.e., flights that were not induced by any obvious goal. The studies on honeybee and wasp homing behavior have shown that landmarks play a decisive role for pinpointing a visually inconspicuous goal and suggest that the animals may even generate specific optic flow patterns, helping them to detect nearby landmarks (e.g., Lehrer, 1993; Zeil, 1993a,b). These findings raise the question, whether information about landmarks positioned around a goal is reflected in the responses of motion sensitive neurons during learning flights in bees. This issue has not been addressed so far, although important groundwork was provided by studies successfully characterizing neurons in the visual motion pathway of both honey- and bumblebees (Ribi, 1975; Ribi and Scheel, 1981; DeVoe et al., 1982; Ibbotson and Goodman, 1990; Ibbotson, 1991a,b; Paulk et al., 2008; Hung et al., 2011, 2013). Therefore, we combine here behavioral and electrophysiological methods to investigate in the context of spatial navigation whether and how bumblebee motion sensitive neurons represent landmark information.

## Materials and methods

Koppert (Berkel en Rodenrijs, the Netherlands) provided commercial bumblebee hives that we kept in custom-built Perspex boxes at a day/night cycle of 12 h. The temperature was kept between 23 ± 2°c at 50% relative humidity. Exclusively bumblebee workers (body length 1.5 ± 0.3 cm) were used for the experiments.

### Behavioral experiments

We let bumblebees (*Bombus terrestris*) enter a circular flight arena (diameter: 1.95 m) that was lined with a Gaussian-blurred red/white random dot texture on walls (height: 50 cm) and floor. The bees were trained to find a see-through Perspex feeder (height: 10 cm) providing a sugar solution between three red cylinders that acted as landmarks (height: 25 cm, diameter: 5 cm). To test whether the bumblebees used the landmarks to solve the task, we placed the feeder outside the landmark arrangement in control trials. In these cases the bumblebees did not find the feeder, which underlines the role of the cylinders as landmarks. Moreover, the overall landmark constellation was displaced within the arena between learning and return flights without much affecting the time until finding the goal. The details of the behavioral analysis are given in a parallel study (Boeddeker et al., submitted manuscript).

We filmed learning flights at 250 images per second with two high-speed cameras (Redlake motion Pro 500). One camera viewed the flight arena from the side, the other from above, enabling us to reconstruct a 3D flight trajectory afterwards. The complete setup and experimental procedure of the behavioral experiment are described in greater detail in Dittmar et al. (2010) where a similar methodology was used.

### Reconstruction of natural optic flow

We analyzed the movies with a custom-built software (Braun and Lindemann, 2011) using the camera calibration toolbox in Matlab (the Mathworks) to compose a 3D head and body trajectory from the projection of the 3D path in the image plane of the two cameras (for details see Boeddeker et al., 2005; Dittmar et al., 2010).

We assumed a constant head roll angle of 0° and a pitch angle of the bee's head of 24° shifted up relative to the horizontal. These assumptions are based on head angle measurements obtained from the side camera and close-up pictures of the bumblebee head anatomy.

**Figure 1:**
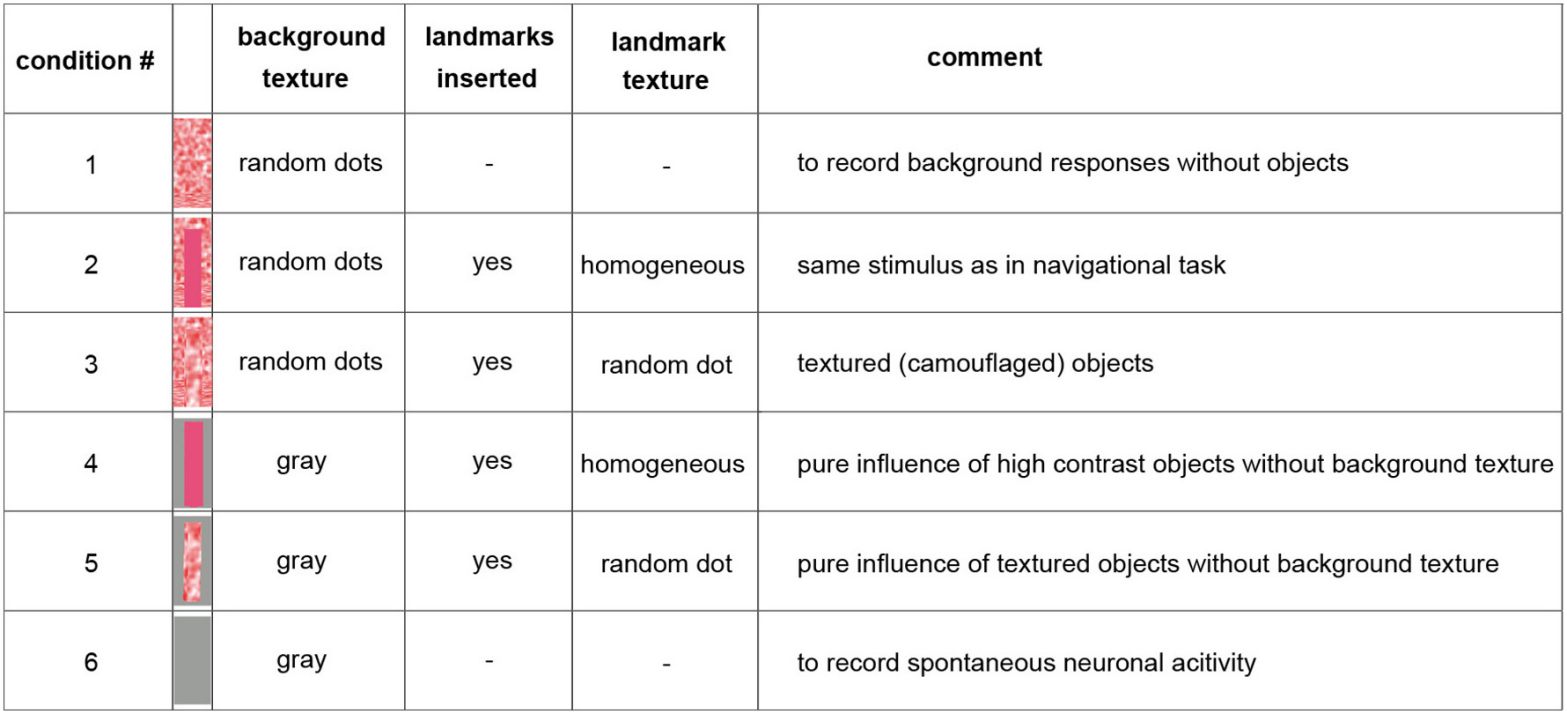
**Overview on the used stimulus conditions**. The inserted pictures show one inserted landmark with the corresponding background texture (arena floor and wall) behind it. The ceiling was kept gray constantly. For more details see Materials and Methods Section. Throughout the main text, we refer to this figure using squared brackets containing numbers that indicate the stimulus condition that was presented, e.g., [condition 2] or [condition 1].

Because of limited recording times and the long duration of the stimulus sequences required for probing the performance of the analyzed neurons, we selected just one learning flight as our basis for analysis. However, we ensured that the landmarks crossed the receptive fields of the analyzed neurons several times during this flight. We fed the 3D-trajectory of this learning flight into a virtual model of the flight arena using custom-built software (Braun and Lindemann, 2011) and determined a panoramic image sequence of what the bumblebee had seen from all the reconstructed positions during this flight [condition 2] (Figure 1). To assess the impact of the of the landmarks and their background as well as of textural features on the neural responses, the flight arena was manipulated virtually in four different ways leading to five different image sequences of about 4.5 s duration: we either left out the objects [condition 1], changed the texture of the objects [condition 3], or changed the texture of the background (walls and floor changed to gray, conditions 4 and 5) (Figure 1). Apart from these manipulations the flight trajectory used for generating the movie was the same. The ceiling of the flight arena was always gray (half-maximum brightness). Under stimulus condition 6 we presented a homogeneously gray screen to the bee. This condition was employed to measure spontaneous neuronal activity (Figure 1).

The head yaw orientation used for stimulus calculation was determined for each frame by the top camera. This was done manually, because automatic tracking of head orientation turned out to be hard to achieve. To validate this manual tracking, we obtained image sequences based on a second, independent measurement of the head trajectory performed by two other persons and found that it did not noticeably affect the neuronal responses. Therefore, we combined the data based on both trajectory versions to a single dataset.

In addition to the reconstructed natural image sequences, we presented bars (10° by 20°) moving horizontally and vertically in both directions at a speed of 100°/s, to approximately determine the size and location of the cells' receptive fields.

### Electrophysiological experiments

With bee wax we glued the back of the thorax onto a small piece of glass, removed the legs and bent the head backwards. Then we glued the bee's head to the edge of the glass without restricting the field of view, but covered the ocelli. Afterwards, we opened the left hemisphere of the head capsule and exposed the lobula.

To ensure the correct placement of the bumblebee within the stimulus device during electrophysiological experiments, we oriented the long axis of the bee's eyes vertically, compensated for roll around the body long axis and centered the animal's head with the antenna bases as points of reference.

We pulled microelectrodes with a Sutter P-1000 puller from aluminosilicate glass pipettes (Harvard apparatus, UK) and inserted them into the lobula of the left brain hemisphere with a heavy micromanipulator (Narashige). Microelectrodes were filled with 1 mol/l KCl and had a resistance range of 40 ± 20 mΩ. As reference electrode a chlorinated silver wire was inserted into a small cut on the other side of the head capsule. The temperature range during the recordings was 30 ± 3°C.

We approached the recording side with the microelectrode from dorsal. We directed the electrodes to the more central areas of the lobula to avoid the risk of recording in the medulla. Given the large receptive field size of the neurons analyzed in our study and the properties of lobula motion sensitive cells characterized in other studies (DeVoe et al., 1982; Ibbotson, 1991b), it is very likely we recorded from the lobula rather than the medulla. We abstained from using saline, because we could ensure that the surface of the brain stayed coated with hemolymph during the entire course of each experiment. In the majority of experiments the reference electrode was placed in the thorax at the position of the animal's legs that were removed. In some cases the reference electrode was placed in a small hole cut into the cuticle of the contralateral brain hemisphere. The location of placement of the reference electrode did not affect our results. We encountered only rarely strong brain movements of the brain as a consequence of pumping activity of the animal. In cases where severe brain movements were observed we stopped recording from the respective bee and proceeded with a new one.

We presented a horizontally moving sinusoidal grating to identify motion-sensitive and direction selective neurons. Despite encountering motion-sensitive neurons that mainly responded with a change of action potential frequency, we focused the search on recordings that showed graded membrane potential changes and that fulfilled the criterion of a stable resting membrane potential.

### Stimulus presentation and data acquisition

For stimulus presentation we used an icosahedral, panoramic LED stimulus device (FliMax) covering most of the visual field of the bee. FliMax has a spatial resolution of 2.3° (for details see Lindemann et al., 2003), which was in a similar range as the spatial resolution of the bumblebee's eye (Spaethe and Chittka, 2003). Stimuli were presented with 8 bits per pixel, allowing 256 different light intensities. We up-sampled the bees' flight trajectory by linear interpolation in time, to be able to replay stimulus movies at 370 Hz, and presented them in pseudo-random order and with an inter-stimulus-interval of 4–6 s. Before each movie started all LEDs were lit for 1 s at half-maximum brightness followed by 0.5 s fading from gray into the first image of the stimulus movie. Since the animal was mounted upside down, we also flipped the stimulus movies along the horizontal axis and shifted the horizon slightly to account for the orientation of the long axis of the bumblebee's eye during flight.

The low-pass filtered responses (2400 Hz cut-off frequency) were sampled at 8192 Hz with a custom build amplifier, digitized (DT 3001, data translation, Marlboro, MA, USA) and stored for offline analysis using Matlab (The Mathworks, Natick, MA, USA).

### Data analysis

Before further analysis, we subtracted the baseline membrane potential from all response traces. As a quality criterion for recordings used for further analysis, we set the minimum range of stimulus-induced membrane potential changes to be at least 2 mv. To account for varying overall response amplitudes of different cells, we normalized the responses. Reference for the normalization of the mean response of a single neuron recorded under different stimulus conditions [conditions 1–6] was the average maximum response of that neuron measured during the stimulus condition that corresponds to the situation during the behavioral experiment [condition 2]. As the different stimulus conditions differ only in one specific feature of the movie while keeping the remaining characteristics the same, it was possible to assess the impact of this feature on the neural performance by subtracting them from each other. The resulting difference trace then represents the response component evoked by targeted environmental feature. To compare the difference traces between different animals we normalized the traces to their peak value for each animal and finally calculated averaged responses of all 27 intersaccades and 26 saccades across different animals.

Correlation coefficients (Pearson's r) were calculated with Matlab's statistics toolbox (Version 7.1, The MathWorks).

## Results

### General characteristics of the recorded neurons

**Figure 2:**
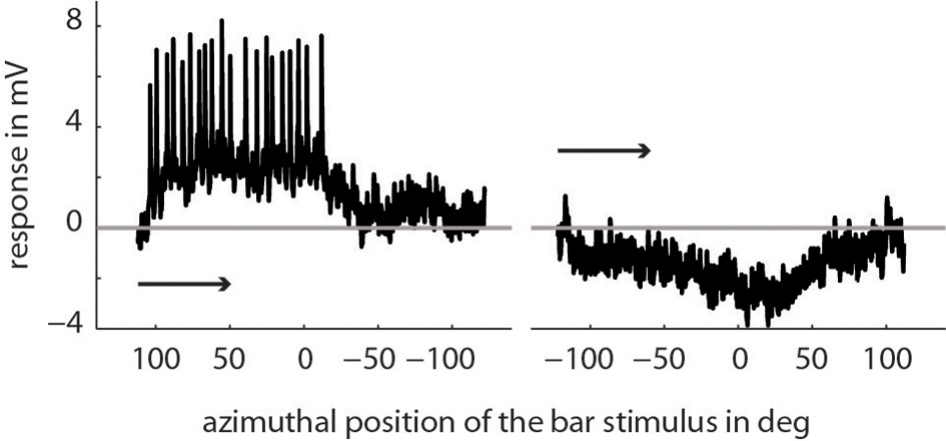
**Example of a single trace recording of a typical LWC sensitive to regressive motion**. Here, we presented a bar moving horizontally through the center of the cell's receptive field (elevation: +20° above horizon) from right to left and vice versa. The x-axis denotes the position of the first (of two) edges of the bar stimulus depending on the direction of movement. As indicated in the Materials and Methods Section the extent of the bar is 10° in azimuth and 20° in elevation and moves with 100°/s. The arrows indicate the direction of movement of the bar.

We recorded the activity of directionally selective motion-sensitive wide-field cells (LWCs) in the bee's lobula. They responded strongest to horizontal wide-field motion and to horizontally moving small bars. Visual stimulation in their preferred direction of motion resulted in graded membrane potential changes partly superimposed by small-amplitude spikes (Figure 2). The resting membrane potential was typically around −50 mV with a stimulus-induced modulation depth of up to 11 mV. Spike-like depolarizations superimposed on the graded response component with up to 35 mV in amplitude were similar to those as described for several types of fly motion sensitive neurons (Hausen, 1982; Haag and Borst, 1997). The horizontal extent of the cells' receptive fields ranged from approximately −10 to 100°, with 0° being in front of the animal, and negative/positive values corresponding to the left and right half of the visual field, respectively. All neurons were most sensitive to motion between angular horizontal positions of 0 and 40° of the visual field. At the temperature of about 30° in our recording setup, a cross-covariance analysis indicated that the time lag between visual input and neuronal response changes was between 21 and 36 ms in different bees.

Based on this functional characterization two functional classes of cells could be characterized: One had a preferred direction from the front to the back, the other from the back to the front. It was not possible to allocate the cells to further subclasses without the use of neuroanatomical techniques or more sophisticated classification stimuli. As the recording time was often limited to a few minutes, we decided to favor a larger number of stimulus repetitions to characterize the cells functionally at the expense of anatomical staining. This allowed presenting 5–20 repetitions of each of the five stimulus conditions.

### Flight behavior of bumblebees

**Figure 3:**
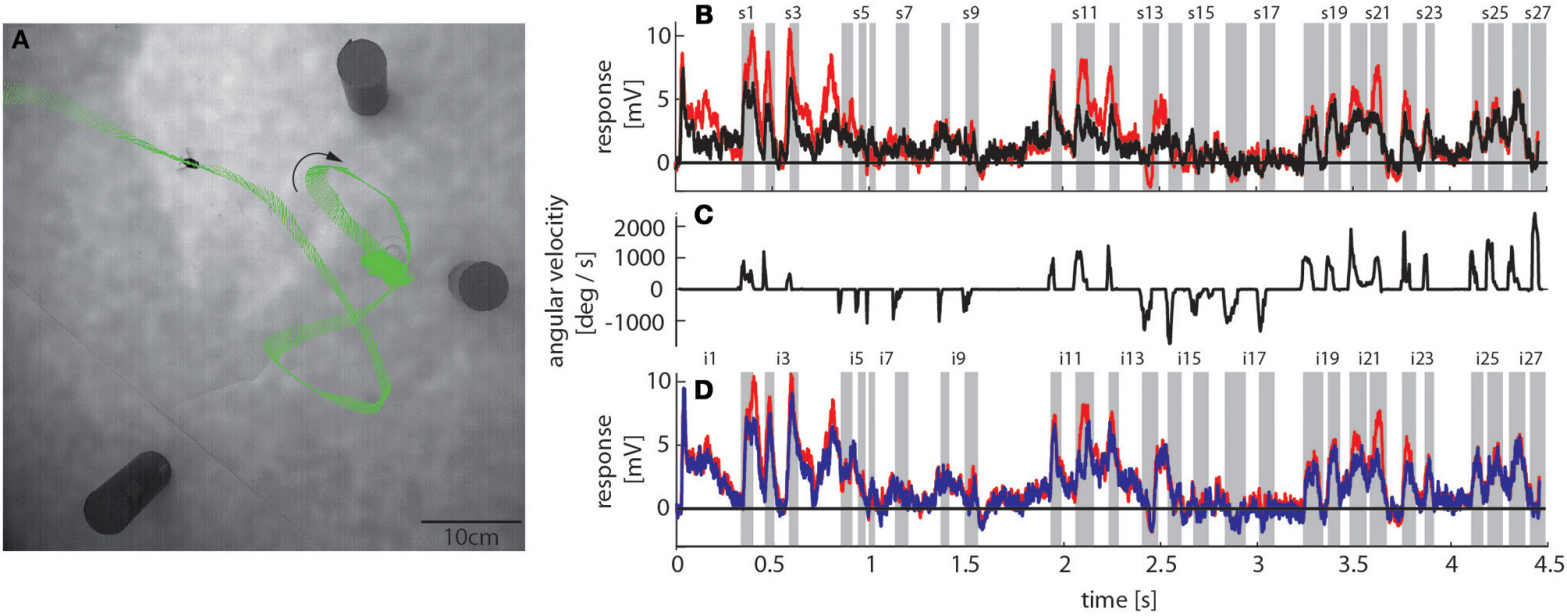
**Bumblebee flight trajectory and corresponding neuronal response traces of a single neuron with preferred direction of motion from back-to-front**. **(A)** Trajectory of a typical learning flight during the navigational task involving landmarks. Each green line indicates a point in space and the corresponding viewing direction of the bee's head each 4ms. The arrow indicates the direction of flight. **(B)** Mean response traces of a single neuron recorded during presentation of two different stimulus conditions. Baseline membrane potential is subtracted. Red trace: homogeneous landmark condition ([condition 2]; 9 repetitions), black trace: no landmark condition ([condition 1]; 7 repetitions). Responses are subdivided into intersaccades and saccades (gray shadings). **(C)** Changes of head orientation. Positive/negative values denote turns to the left and right, respectively. **(D)** Response traces of the same neuron as in **(B)**. Red trace: as described above. Blue trace: random pattern landmark condition, i.e., camouflaged landmarks ([condition 3]; 7 repetitions).

To reconstruct the visual image sequences that are generated by bumblebees during a learning flight we first analyzed their behavior in a local navigation paradigm. We trained them to find a see-through Perspex feeder between three salient objects that acted as landmarks in a textured flight arena. It took trained bumblebees between 4 and 229 s (mean = 40 s, *SD* = 49 s, *N* = 4, 53 flights in total) to find the feeder, which is in a similar range as for honeybees in the same experimental setup (Dittmar et al., 2010). The structure of the bumblebees' learning flight maneuvers was also very similar to that of honeybees (Dittmar et al., 2010) (Figure 3A). In the sample trace shown in Figure 3A the bumblebee started at the feeder and hovered at first in front of it. Then it turned to the left, flew two arcs and finally to the exit of the arena. The fine structure of such flight maneuvers was characterized by a pronounced saccadic separation of translatory and rotatory movements, similar to honeybees and wasps (Boeddeker et al., 2010; Braun et al., 2012; Zeil, 2012). The corresponding time course of the angular velocity around the yaw axis consisted of relatively long phases with only small changes in head orientation (intersaccades), meaning that almost pure translational optic flow was perceived. These phases were interspersed by saccades with fast rotations of head and body around the yaw axis (Figure 3C). This gaze strategy facilitates the acquisition of motion cues for distance estimation (Egelhaaf et al., 2012).

Based on these behavioral data we ask whether information about the landmarks surrounding a barely visible goal is reflected in responses of motion sensitive visual interneurons.

### Are the landmarks represented in the neuronal responses?

To investigate the influence of landmarks on the responses of motion sensitive lobula wide-field cells (LWCs) we reconstructed the visual input during a typical learning flight of a bumblebee (Figure 3A), and presented this ego-perspective movie, while the bee was tethered in the center of our panoramic LED stimulus device. Additionally to the original optic flow sequence we presented several systematic alterations to study how different landmark properties are reflected in the neuronal responses (see Materials and Methods Section).

How is this naturalistic visual input reflected in the neuronal responses? LWCs in the bee are known to respond in a direction-selective way to visual motion (DeVoe et al., 1982; Ibbotson, 1991b; Paulk et al., 2008). In our account we focused on one functional class of LWCs that is direction-selective to horizontal motion, with a preferred direction from back-to-front. These cells responded to visual stimulation with graded membrane potential changes partly superimposed by spikes (Figure 2), similar as depicted in DeVoe et al. (1982). This response mode is also typical for motion sensitive visual interneurons in the lobula plate of the fly (Hausen, 1982; Haag and Borst, 1997; Egelhaaf, 2006).

To assess how objects affect neuronal activity, we compared the responses elicited by the original stimulus movie [condition 2] (red trace in Figure 3B) to responses elicited by the same movie, but without landmarks [condition 1] (black trace in Figure 3B). The original movie corresponded to the visual input that was experienced by a freely flying bumblebee during a learning flight (Figure 3A). In the 11 neurons that we could analyze we found that the two response curves differed considerably in some sections of the flight revealing a strong object influence on the neuronal response (Figure 3B). In contrast, the response traces obtained under the homogeneous object condition [condition 2] (red trace in Figure 3D) were much more similar to the responses evoked when the object had the same texture as the background [condition 3] (blue trace in Figure 3D).

**Figure 4:**
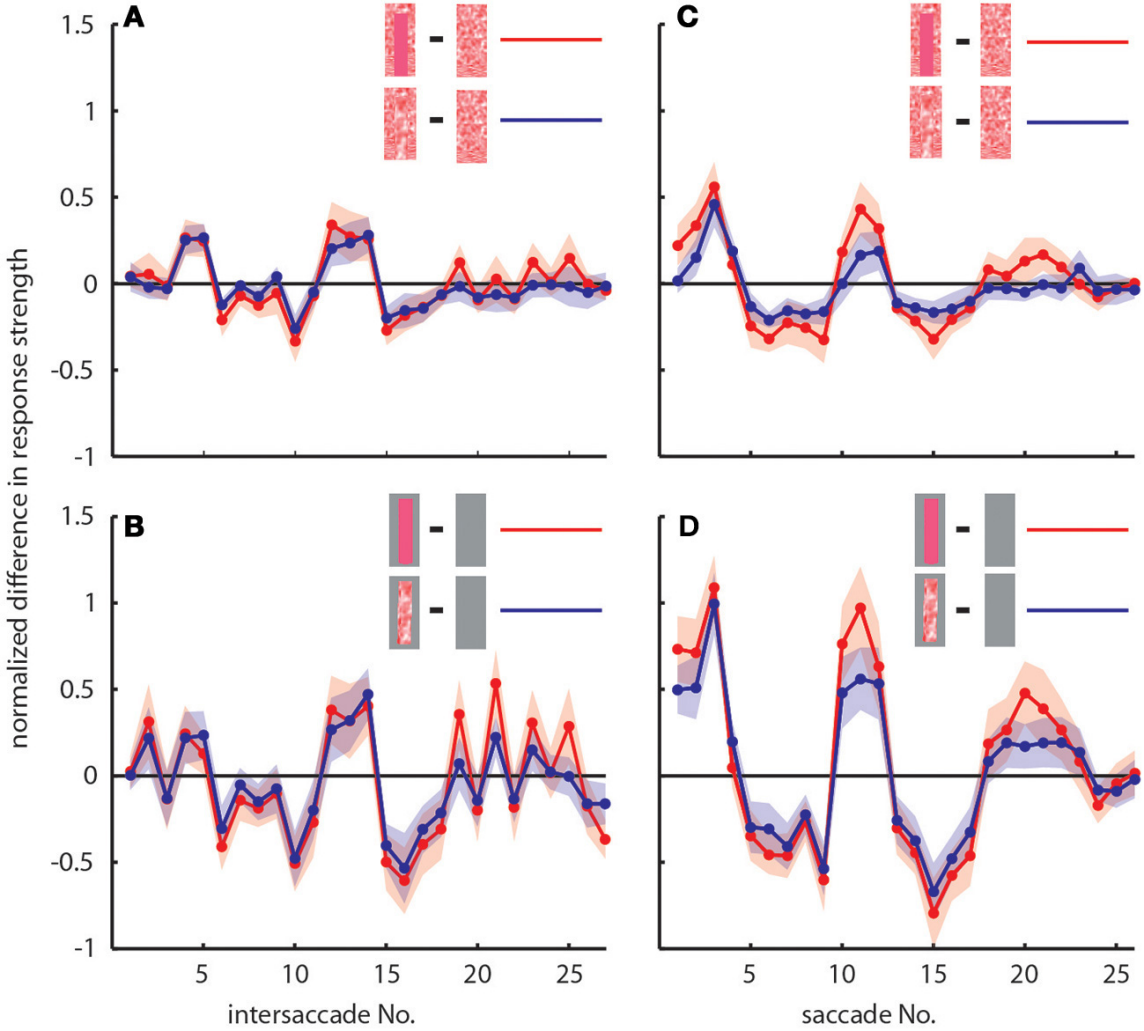
**Response profiles of mean, normalized differences between pairs of stimulus conditions for consecutive intersaccades (A,B) and saccades (C,D) during the learning flight of LWCs with a preferred direction from back-to-front**. **(A,C)** Mean normalized difference of responses to one stimulus condition with object and one without (“object-induced response changes”). Red traces: object-induced response change for “homogeneous landmark” [2] and “no landmark” condition [1]. Blue traces: object-induced response change for “random pattern landmark” condition [3] and “no landmark” condition [1]. For **(A)** and **(C)** the number of included cells is *n* = 11. **(B,D)** Traces have the same meaning as in **(A)** and **(C)**, but object-induced response changes were induced by stimulus conditions with the random background being replaced by a uniform gray background. For **(B,D)**, *n* = 7. Shadings indicate SEM. For details on normalization procedure see Materials and Methods.

To determine the differences between neuronal response traces recorded under different stimulus conditions we averaged, separately for each saccade and intersaccade, the normalized difference of the responses to the stimulus conditions without objects [condition 1] and with objects that carried a different texture [conditions 2 and 3]. Values that substantially deviated from zero indicated response changes caused by the objects. Depending on the stimulus condition we were able to record from 7 and 11 neurons (the same 7 neurons plus 4 additional neurons). We observed a characteristic temporal pattern in the profile of object-induced response changes that was similar between intersaccades (Figure 4, left side) and saccades (Figure 4, right side). This pattern also remained when we artificially removed the background pattern to exclude background effects and to just let the objects influence the neuronal responses (Figures 4B,D). These general similarities of the intersaccadic and saccadic object-induced response profiles underline that the neuronal responses were shaped by the spatial layout of the environment. Therefore, LWCs do not only perceive wide-field motion, but their responses during a learning flight also convey information about the landmarks, which are important for bees to be able to find their goal during local navigation.

### Neuronal responses to camouflaged landmarks

Bees can use camouflaged landmarks that carry the same texture as the background for homing, and the search time for such objects is similar to that seen for high-contrast landmarks (Dittmar et al., 2010). Since these camouflaged landmarks are hard to detect in stationary images, it was suggested that bees might use relative motion cues—present in optic flow during translatory flight phases—to perceive them (Dittmar et al., 2010). Therefore, we tested whether landmarks that can only be discriminated by relative motion cues are reflected in the responses of LWCs. To this end, we camouflaged the objects by using the same random dot texture for the objects and the background [condition 3], i.e., floor and walls.

In accordance with the characteristics of goal-finding behavior (Dittmar et al., 2010) the object-induced intersaccadic responses did not differ much between objects with random dot texture and homogeneously red texture (Figure 4A). To confirm the object influence on the neuronal responses with a less complex stimulus, we also presented stimuli with both versions of object texture (red texture and random dot texture), but with arena wall and floor being plain gray [conditions 4 and 5]. In this way the background could not affect the neural responses, leaving just the objects to shape them. Under these conditions the profile of object-induced response changes was similar to those profiles obtained with a textured background (compare Figures 4A,B). The only prominent difference was the larger modulation depth of the profile under the conditions without background (Figure 4B). This difference can potentially be attributed to different contrast values between background and object. The similarity of the resulting intersaccadic profiles of object-induced response changes corroborates the above conclusion: During learning flights the intersaccadic neural responses provide information about the spatial arrangement of landmarks in the vicinity of the goal.

**Figure 5:**
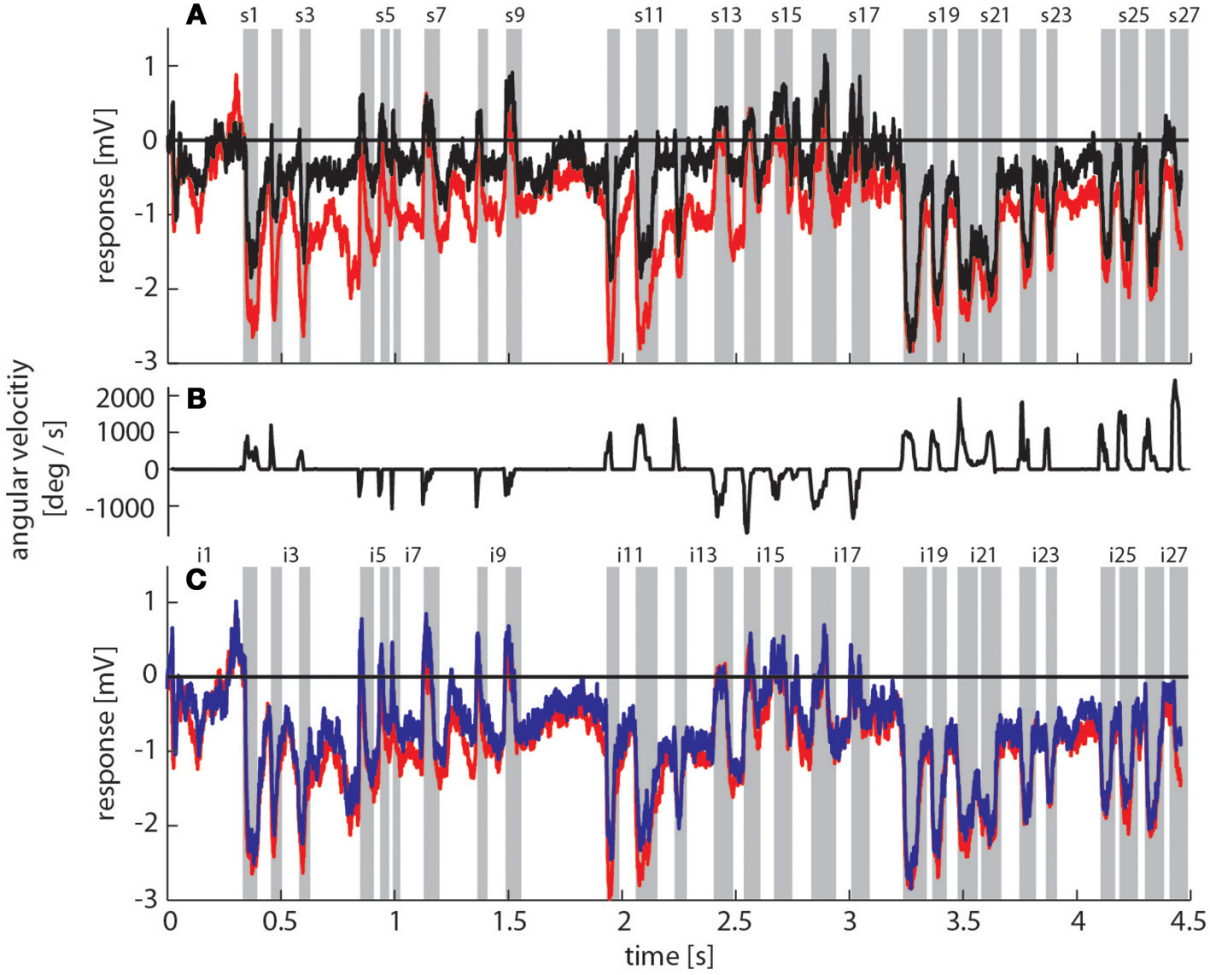
**Response traces of a single LWC sensitive to front-to-back motion during presentation of different stimulus conditions**. **(A)** Response traces of one neuron recorded during visual stimulation. Baseline membrane potential is subtracted. For the evaluation we calculated the mean responses over many trials (also Figure 1B). Black trace: no landmark condition ([condition 1]; 6 repetitions), red trace: homogeneous landmark condition ([condition 2]; 9 repetitions). Neuronal response is subdivided into intersaccades and saccades (gray shadings). **(B)** Changes of head orientation. Positive/negative values denote turns to the left and right, respectively. **(C)** Response traces of the same neuron as in **(A)**. Red trace: homogeneous landmark condition ([condition 1]; 9 repetitions), Blue trace: random pattern landmark condition, i.e., camouflaged landmarks ([condition 3]; 7 repetitions).

**Figure 6:**
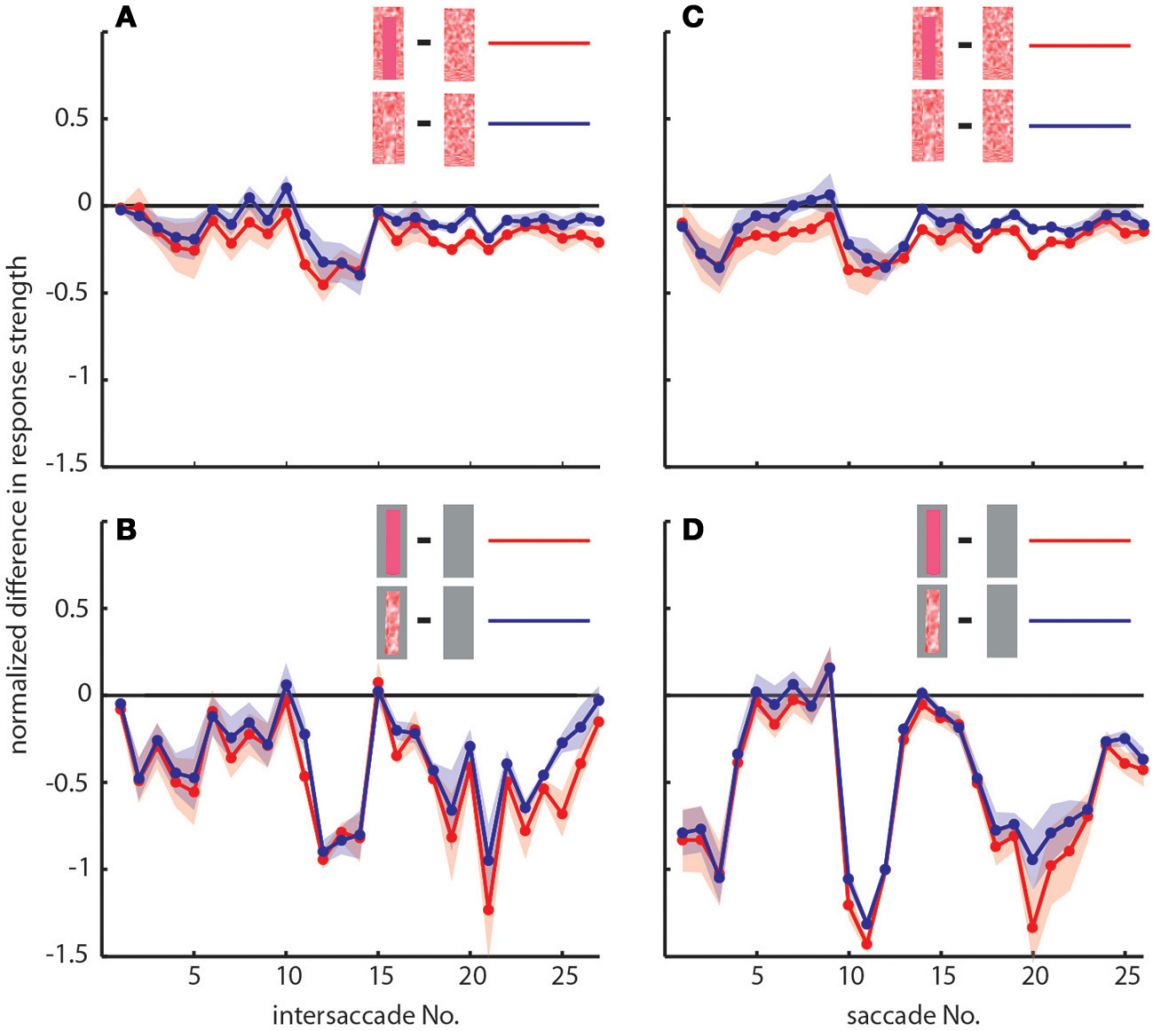
**Response profiles of LWCs with front-to-back as preferred direction of movement**. Plots show object-induced response changes as the mean, normalized differences between two stimulus conditions for intersaccades **(A,B)** and saccades **(C,D)**. For **(A)** and **(C)** traces indicate object-induced response changes for subsequent intersaccadic **(A)** or saccadic **(C)** intervals. Red traces: object-induced response changes during “no landmark” condition [1] subtracted from those during “homogenous landmark” condition [2]. This indicates the influence of homogeneously textured objects. Blue traces: object-induced response changes during “no landmark” condition [1] subtracted from those during “random pattern landmark” condition [3]. This indicates influence of objects that were randomly patterned. For **(B,D)** traces have same meaning, but responses were obtained during stimulus conditions with a gray background. Number of cells included in this figure is *n* = 3. Shadings indicate s.e.m. All responses were normalized to the cells' maximum mean response during intersaccades or saccades, respectively, during the stimulus condition identical to behavioral situation (homogeneous landmark condition, [1]). For more details see Materials and Methods Section.

We obtained similar results also for a second functional class of LWCs, which we recorded less often and that had an opposite preferred direction of motion, i.e., from front-to-back (Figure 5). Again, the responses under the original stimulus condition with homogenous landmarks [condition 2] (red trace in Figure 5A) are larger during most of the flight trajectory than the responses obtained without objects [condition 1] (black trace in Figure 5A). Moreover, the responses evoked with homogenous landmarks [condition 2] (red trace in Figure 5C) are much more similar to those obtained with textured landmarks [condition 2] (blue trace in Figure 5C). Hence, these cells showed very similar general properties like back-to-front LWCs apart from the difference in their preferred direction of motion. Also the profiles of object-induced response changes were similar for these two classes of cells for landmarks with different textures (Figure 6). Depending on the cells' preferred direction of motion, the deviations from zero were approximately phase-inverted to those obtained in LWCs with back-to-front motion as preferred direction (compare Figure 6 and Figure 4).

### Relation between landmark-induced responses and flight maneuvers

**Figure 7:**
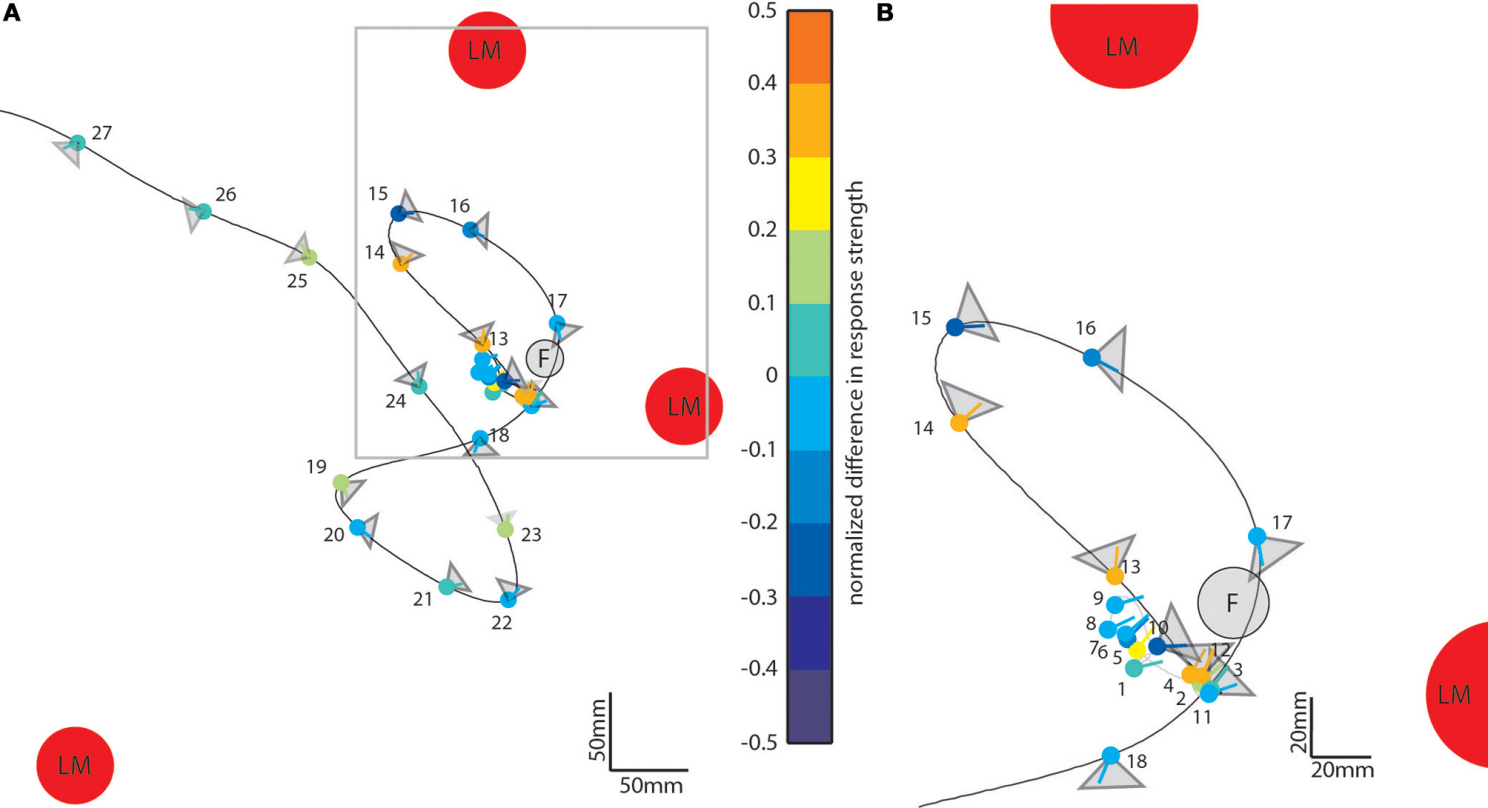
**Object-induced intersaccadic response changes of a LWC with a preferred direction from back-to-front plotted along the flight trajectory of the replayed ego-perspective flight sequence (compare to Figure 2A)**. This example indicates the difference between the no-landmark condition [condition 1] and the homogeneous landmark condition [condition 2]. Red circles denote landmarks (LM) and the gray circle the see-through feeder (F). The color-coded dots represent the position of the bee in the middle of each intersaccade; the corresponding line shows the direction of view. Warm colors indicate response increments, and cold colors response decrements induced by objects in the receptive field of the cell. The gray triangles attached to each dot illustrate the typical horizontal extent of the receptive field of the analyzed cells (*n* = 11). **(A)** Overview on entire learning flight. **(B)** Enlarged view on the intersaccades during the beginning of the learning flight. For sake of clarity the receptive field areas are not indicated for intersaccades 1–9.

So far, we have seen that landmarks affect the neural responses during different sections of the flight. To assess what kind of maneuvers during the learning flight lead to prominent object-induced response changes, we averaged the intersaccadic object-induced response level and plotted the mean response along the original flight trajectory using a color code (Figure 7). The color-coded dots indicate the position of the bee in the middle of the intersaccades (indicated by their number); the lines give the viewing direction of the bee and the triangles the orientation and angular width of the analyzed cells' receptive field. The color code of the dots reflects the mean intersaccadic response difference between the homogenous landmark condition [condition 2] and the “no landmark” condition [condition 1] (see also Figure 4A; red trace). The data are shown for our larger dataset, from LWCs with preferred direction of motion from back-to-front (*n* = 11).

During forward flight, i.e., when the viewing direction roughly coincides with the direction of the flight trajectory (see e.g., intersaccades 15–18) the object responses did not deviate much from zero for most of the time, with only a slight negative shift (Figure 4, e.g., intersaccades 15–18). This negative shift was the consequence of the objects moving through the receptive field (Figure 7, gray triangles) from front-to-back, i.e., in the anti-preferred direction of the cells. Strong deviations from zero were generated when the bee translated with a larger sideways than forward velocity, i.e., when the viewing direction deviated much from the direction of the flight trajectory (e.g., intersaccades 12–14). As a measure of the relation between both velocity components we took the angle α between flight direction and viewing direction for the 27 analyzed intersaccadic intervals. We found a positive correlation between the intersaccadic neuronal responses and the angle α. The correlation values amounted to *r* = 0.38 (*p* < 0.05) for the responses to the homogeneous red landmarks in front of the textured background [condition 2]. The correlation values were even higher (*r* = 0.52; *p* < 0.01) for the responses to the stimulus condition with the landmarks being textured with random dots [condition 3]. The correlation values were similar when correlating α with the intersaccadic responses during the stimulus conditions with gray background (condition 4: *r* = 0.41, *p* < 0.05; condition 5: *r* = 0.52, *p* < 0.01). The large sideways movements caused a specific optic flow pattern on the retina: Landmarks close to the animal moved faster than the background, leading to enhanced neural responses during stimulus conditions with objects (see Figure 1: Conditions 2 and 3 compared to condition 1 as well as condition 4 and condition 5 compared to condition 6).

For other parameters than the angle α, we did not find pronounced correlations with the intersaccadic neural responses. The retinal size of the landmarks within the cell's receptive field was not correlated significantly with the normalized object-induced intersaccadic response changes (random dot pattern background: *r* = −0.10; *r* = −0.14; gray background: *r* = −0.19; *r* = 0.20). Similarly weak correlations are obtained when we only took into account the largest landmark in the visual field or the overall size of all landmarks within the receptive field. Moreover, the distance to the landmarks in the receptive field to the eye did not influence the intersaccadic neural response significantly (*r* = −0.09 to 0.09), despite the distance dependence of the retinal velocity. This finding is the likely consequence of more than one stimulus parameter, such as the velocity of retinal pattern displacements or the direction of motion and pattern contrast, influencing the response strength of LWCs. In conclusion, among the different stimulus parameters we found the angle α and, thus, the relation between sideways and forward velocity to be the most important determinant of object influences on the neuronal responses.

**Figure 8:**
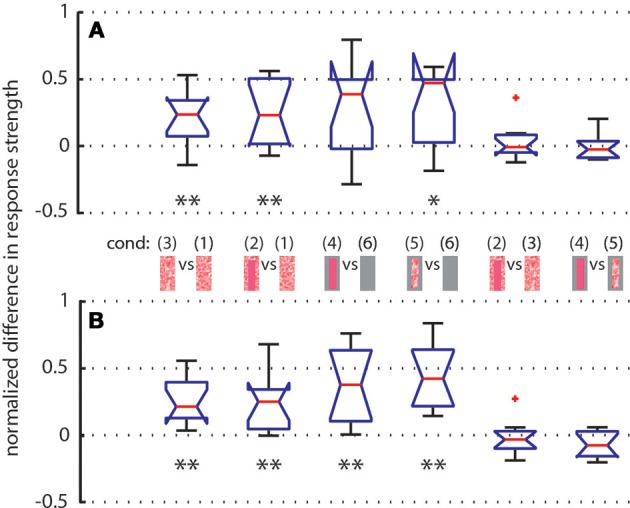
**Normalized differences between responses of LWCs to pairs of stimulus conditions (see pictograms between A and B) during intersaccade No. 13 (subplot A) and No. 14 (subplot B) Depicted data from LWCs with preferred direction of motion from back-to-front**. Number of cells: *N* = 11. Explanation of box symbols: red central horizontal line—median; box edges represent 25th and 75th percentiles; whiskers—most extreme data points that are not outliers (>75th percentile +1.5^*^ box size OR <25th percentile –1.5 ^*^ box size). Outliers are plotted separately (red dots). Notches describe the 95% confidence intervals of the median. Two medians are significantly different at the 5% significance level if the notches do not overlap (McGill et al., 1978). Asterisks indicate statistically significant deviation from zero (two-tailed *t*-test; ^*^p < 0.05, ^**^p < 0.01). Median at zero level means no object influence.

To further quantify the object-induced response changes, we selected two adjacent intersaccadic intervals (no. 13 and 14) with clear object-induced responses. Here, the bee moved to the left side and thus allowed one of the objects to move on the retina in the cell's preferred direction. The object-induced response changes elicited by camouflaged landmarks [condition 3] were very similar to those induced by the homogeneous red landmarks [condition 2]. For both analyzed intersaccades the object led to significant response deviations from zero (Figure 8). Similar deviations could be demonstrated for the gray background condition (Figure 8). These findings corroborate quantitatively the above conclusion that the objects significantly influence the response of the recorded cells.

We analyzed how object texture affects the neural response changes during the two selected intersaccades by comparing the responses to stimulus conditions with the same background texture but different object textures. We did not find significant deviations from zero (Figure 8), indicating that the texture of objects is not strongly reflected in the neural response, as already suggested by the almost equal strengths of the intersaccadic response profiles for different object textures (Figure 4).

In summary, these findings further support our conclusion that the object-induced response does not strongly depend on object texture during in the intersaccadic parts of the neuronal responses.

### Texture effects on neuronal responses during saccadic flight phases

Object-induced neuronal response changes were also evoked during saccades (Figures 4C,D, 6C,D) and, thus, did not only affect the intersaccadic neuronal responses. Even the camouflaged objects had an impact on the neuronal response during saccades, although the translatory retinal motion component can be expected to be much smaller than the fast rotatory component (Figures 4C,D). The object-induced differences during saccades were similar to the ones observed during the intersaccades (Figures 4A,B, 6A,B). This finding is likely to be the consequence of subsequent saccades and intersaccades occurring at similar locations in the flight arena and the bee's residual translatory locomotion that is due to the fact that the bee is continuing to fly along its arc, without stopping for each single saccade. Thus, the retinal input during saccades might be affected by the environment in similar way (as e.g., Figure 4).

In contrast to the intersaccades, the responses evoked during the saccades were affected by the retinal size of the landmark. We found a positive correlation for back-to-front LWCs between the retinal size of the objects and the saccadic responses when the background was randomly textured (for condition 2, *r* = 0.36; for condition 3, *r* = 0.28). These correlations were stronger when the background was gray and, thus, the neural response was affected exclusively by the objects (for condition 4: *r* = 0.47; for condition 5: *r* = 0.43).

The object influence on the saccadic responses depended also slightly on the texture of the objects (Figures 4C,D). With gray background [conditions 4 and 5] the object texture influence was stronger. These effects were also visible for front-to-back LWCs (Figure 6), although in a less pronounced way.

## Discussion

We show that direction-selective motion sensitive wide-field cells (LWCs) in the lobula of bumblebees convey information about the spatial arrangement of landmarks that help bees localizing a hardly visible goal location. It is long known that bees use motion cues in a variety of other behaviors like landing, pattern discrimination and in the determination of traveled distances (Lehrer and Collett, 1994; Kern et al., 1997; Srinivasan and Zhang, 2000, 2004, Srinivasan et al., 2000a,b; Lehrer and Campan, 2005; Wolf, 2011). In this account we focused on learning flights as fundamental components of navigation behavior. We analyzed whether visual landmarks surrounding a goal location are represented in the bee's visual motion pathway using behaviorally relevant, naturalistic visual stimulation in electrophysiological experiments. The temporal profiles of responses of LWCs during both intersaccades and saccades are affected by the landmarks surrounding the barely visible goal, indicating that the neuronal responses of LWCs contain information about the spatial layout of the environment.

Similar to other insects like, e.g., various fly species, wasps and ants (Land, 1999; Schilstra and van Hateren, 1999; van Hateren and Schilstra, 1999; Mronz and Lehmann, 2008; Braun et al., 2010; Geurten et al., 2010; Lent et al., 2010; Kern et al., 2012; van Breugel and Dickinson, 2012; Zeil, 2012), bees separate their locomotion into phases of saccades and intersaccades (Boeddeker et al., 2010, submitted manuscript; Boeddeker and Hemmi, 2010; Braun et al., 2012). During the intersaccadic phases of translatory motion bees can gather depth information from the environment as has been shown before for blowflies (Boeddeker et al., 2005; Kern et al., 2005, 2006; Karmeier et al., 2006; Egelhaaf et al., 2012; Hennig and Egelhaaf, 2012; Liang et al., 2012). However, in these analyses of blowflies only spontaneous flights could be considered and objects were introduced virtually in most of these studies after the behavioral experiments had been performed, just for stimulus generation. Hence, the functional significance of these objects was not clear. In contrast, we analyzed here how behaviorally relevant landmarks are represented during a learning flight in the context of spatial navigation of bumblebees. We found that the activity of two types of LWCs is modulated by landmarks during the intersaccadic phases of a learning flight, but also during saccades. Whereas textural differences of objects influence the neuronal responses during saccades, they do not differ significantly for differently textured objects during intersaccades.

This independence of intersaccadic LWC responses from object texture mirrors the overall performance of bees in local navigation behavior that was largely unaffected by textural changes of landmarks (Dittmar et al., 2010). Rather than by object texture, response modulations during self-motion of the animal seem to be caused by factors like the relative motion of the object against the background. Especially during sideways flight maneuvers, we found object-induced neuronal responses. Hence, independent of the actual mechanisms that induce object-driven response changes in LWCs we conclude that during intersaccades the bee might be provided with information related to the geometrical layout of the immediate surroundings in which it is navigating.

How could an animal exploit the information provided by the neural responses induced during saccades? An earlier study (DeVoe et al., 1982) suggested that LWC responses might be involved in the animal's optomotor response. Given the response differences between both landmark textures during saccades, they might also provide signals that can help extracting textural information about the environment. This information could, at least in principle, be used to distinguish landmarks carrying different textures. Honeybees have been shown to use this textural information, when it provides positional information in a navigation task (Dittmar et al., 2011).

We coarsely classified the recorded LWCs into two groups according to their preferred direction of motion and still find a characteristic correlation between the landmark-induced response components and the amount of sideways locomotion during intersaccadic intervals. We thereby possibly pooled the data across several cell types with similar but slightly different response properties by using the preferred direction as the main criterion for cell classification. Analyzing unique cell types individually would potentially have led to even more pronounced effects compared to what we already observe while possibly averaging across a population of LWCs. Nevertheless, our results indicate that we describe a global response property of such a population of LWCs that is not tied to a single cell type. For both classes of cells, front-to-back and back-to-front LWCs, we show that landmarks appearing in the receptive field modulate the cells' membrane potential and therefore convey information about the animal's surroundings. We do not know whether we recorded the mixed graded and spiking responses in the dendritic regions of LWCs or in large diameter axons with large length constants. However, the transfer of information by LWCs via graded modulation of the membrane potential or a combination of graded membrane potential changes and spikes is very common in insect neurons even in presynaptic areas of neurons with large-diameter axons, indicating that both signal components may be transferred to postsynaptic neurons (Haag and Borst, 1997; Warzecha et al., 2003; Beckers et al., 2007, 2009; Rien et al., 2011). The population response provided by different LWCs could then be used in the context of local homing to compare the current LWC activity profile to a previously stored LWC activity profile—the neural correlate of an optic flow snapshot of a location.

We recorded from neurons in the lobula, which is a predominantly visual processing stage before the information is distributed into brain areas that are involved in pronounced multimodal processing and learning (Hertel and Maronde, 1987; Hertel et al., 1987; Paulk and Gronenberg, 2008; Paulk et al., 2009; Mota et al., 2011). Most likely, the visual input to the lobula is processed independently from other sensory modalities. Recent studies showed, however, for flies that an active behavioral state of the animal affects the amplitude of neuronal responses and may even somewhat shift their velocity tuning (Chiappe et al., 2010; Maimon et al., 2010; Rosner et al., 2010; Jung et al., 2011). Such effects of an active behavioral state would probably not affect our conclusion that landmarks indicating a goal location are reflected in the intersaccadic neural responses during learning flights. Rather, an increasing gain of the neurons might make it even easier for the animal to distinguish the landmarks from their background.

It would be interesting to know where the LWCs we recorded from project to, as a multitude of behavioral studies show that bees store, compare and also combine multisensory cues for navigation. Due to often very short recording times we decided to not stain the neurons, although this would have allowed us to individually identify them and their connections anatomically. LWC projections have been reported from to brain regions like the contralateral lobula (DeVoe et al., 1982) or the mushroom body calyx (Paulk and Gronenberg, 2008) that is known to play an important role for multimodal learning. Additionally, it has been shown recently that visual novelty during learning flights leads to up-regulation of an immediate early gene in the mushroom bodies of honeybees (Lutz and Robinson, 2013), indicating the involvement of the mushroom body into visual learning processes. Moreover, the lobula is connected to the central complex (Ribi and Scheel, 1981; Paulk et al., 2008), an area that may also be involved in multimodal processing (Gronenberg, 1986; Maronde, 1991) and was demonstrated to be involved in processing visual information in bees (Milde, 1988) and in place learning by *Drosophila* (Ofstad et al., 2011). The functional properties of the recorded neurons and, in particular, the responses to objects do of course not depend on their anatomical characterization. Still, anatomical evidence on the up- and downstream connections could provide further insights on the neuronal substrate of homing behavior and should be obtained in follow up studies. Nonetheless, even without knowing the projection area of these cells, we show for the first time with naturalistic stimulation that they convey information about the spatial layout of the landmark configuration—information that is crucially relevant in a homing context.

### Conflict of interest statement

The authors declare that the research was conducted in the absence of any commercial or financial relationships that could be construed as a potential conflict of interest.
